# Elective Orthopaedic and Trauma Patients in Southern Italy are Vitamin D Deficient. A Pilot Study

**Published:** 2018-03-31

**Authors:** Antonio Foccillo, Rocco Aicale, Nicola Maffulli

**Affiliations:** 1 Department of Musculoskeletal Disorders, School of Medicine and Surgery, University of Salerno, Salerno, Italy; 2 Mary University of London, Barts and the London School of Medicine and Dentistry, Centre for Sports and Exercise Medicine, Mile End Hospital, 275 Bancroft Road, London E1 4DG, England

**Keywords:** Arthroplasty, knee, hip fracture, vitamin D

## Abstract

Many patients present with Vitamin D (Vit d; 25 (OH) D) deficiency on admission to hospital. The present study evaluated the levels of serum concentration of 25 (OH) D in patients undergoing elective arthroplasty of the hip and knee and in patients with proximal femur fracture. A total of 90 patients of both sex were recruited, 20 with osteoarthritis of the knee, 29 with osteoarthritis of the hip, and 41 with a hip fracture. The levels of Vit D were measured twice: on admission and on the fourth day after surgery. In patients undergoing knee arthroplasty, the levels of Vit D were 16.85 ng/ml in the preoperative and 18.235 ng/ml in the postoperative period. In patients undergoing hip arthroplasty, we found levels <4.20 ng/ml for men, while in women 25.20 ng/ml pre-operatively and 22.8 ng/ml post-operatively. In patients with a hip fracture, men progressed from very low levels preoperatively (6.38 ng/ml) with post-operative levels of 18.44 ng /ml. Women exhibited a slight improvement from 12.24 ng/ml pre-operatively to 17.9 ng/ml postoperatively. Patients who are candidates for hip and knee arthroplasty and in hip fracture patients exhibit low Vit D levels. The activation of the inflammatory cascade may induce reduction of Vit D levels, and this fall is associated with impaired bone health.

## I. INTRODUCTION

Vitamin D has been appreciated for its role in calcium homeostasis and bone health since its identification in 1921 [1]. It’s a fat soluble vitamin that regulates calcium metabolism, promoting its re-absorption in the kidney [2]. The dosage of 25 (OH) D in the serum is the most accurate method to estimate its concentration in the body [3]. A very small proportion of 25 (OH) D remains free or unbound in plasma (0:02 to 12:05%) and represents the metabolically active one [2].

Around 80–90% of Vit D is bound to circulating vitamin D-binding protein (VDBP), and 10–20% is bound to albumin. In Italy, a level less than 30 ng/ml is considered insufficient [4,5].

Vitamin D consists of 2 bioequivalent forms: D2 (or ergocalciferol) obtained from dietary vegetable sources and oral supplements, D3 (or cholecalciferol) obtained primarily from skin exposure to ultraviolet B (UVB) radiation in sunlight, ingestion of food sources such as oily fish and variably fortified foods (milk, juices, margarines, yogurts, cereals, andsoy), and oral supplements. Both D2 and D3 are biologically inert [1].

Once absorbed from the intestine, they are metabolized in the liver to 25-hydroxyvitamin D [25(OH)D], composed of 25(OH)D2 and 25(OH)D3; 25(OH)D (also called calcidiol) is subsequently converted to 1,25-dihydroxyvitamin D [1,25(OH)2D], also known as calcitriol, in the kidney and select other tissues by the action of the 1α-hydroxylase enzyme.

Vitamin D is a critical hormone for many events, not only on bone growth and remodeling, but also on immune function and other metabolic pathways involved in the healing process [6].

A prospective analysis of 81 patients in the Northeastern (USA) undergoing elective orthopedic surgical procedures showed that two-thirds of this adult population had low vitamin D levels ( < 30 ng/mL) [7].

Several reports of hypovitaminosis D being associated with a poor preoperative functional state in patients undergoing knee arthroplasty, but the effect of low vitamin D on postoperative function is less clear [8,9].

Lavernia et al. [10] demonstred that vit D insufficiency has also been linked to poor functional outcomes after hip replacement. They retrospectively analyzed 60 consecutive patients who underwent elective primary total hip arthroplasty and found that patients with vitamin D levels less than 30 ng/mL had lower preoperative Harris Hip Scores (HHS) as compared to patients with vitamin D levels greater than 30 ng/mL [10].

Many elderly patients with hip fractures are anthropometrically malnourished [11]. Malnutrition in ageing is common, and this is due to a progressive a loss of appetite, a reduction in food intake and also to vitamin D deficiency, largely due to skin atrophy [12].

Furthermore, low vitamin D, in association with high parathyroid hormone levels, has been found to increase the risk of muscle wasting in old age [13–15]. Furthermore nutritional status was not related to post-operative complications after hip fracture treatment [11].

There is a paucity in literature about the prevalence of hypovitaminosis D in groups of patients undergoing elective arthroplasty of the hip and knee, and in patients with hip fractures. We wished to ascertain whether these patients were hypovitaminosis, and whether there were differences in serum levels of Vitamin D between these three groups and subgroups of patients.

## II. MATERIALS AND METHODS

The study was conducted at the Department of Orthopedics and Traumatology of the University Hospital San Giovanni di Dio e Ruggi d’Aragona and all records were retrieved from the hospital database between April 2015 to October 2016. We included all patients with osteoarthritis of the knee and the hip, and all patients admitted with traumatic proximal femur fracture.

The serum levels of 25 (OH) D were measured using ADVIA Centaur Vitamin D Total assay (Siemens Healthcare Diagnostics Inc, Tarrytown, NY), a competitive immunoassay in 1-Step, with an incubation type of 18 minutes, which uses a murine monoclonal antibody anti-fluorescein covalently linked to paramagnetic particles (PMP), a murine monoclonal antibody anti-25 (OH) vitamin D labeled with acridinium ester (AE), and a vitamin D analogous marked with fluorescein, in the serum of these patients.

We classified hypovitaminosis D on the basis of the measurement of serum 25(OH)D concentrations, as recommended by Lips [16] . Concentrations of 10–20, 5–10, and < 5 ng 25(OH)D/mL were classified as mild, moderate, and severe hypovitaminosis D, respectively [17].

All records were entered using Microsoft Excell software. The levels of Vit D were measured in two times: on the admission, and on the fourth day after surgery. Following the index operation, patients received Vit D supplementation therapy with DIBASE (2500 IU/2.5mL).

## III. RESULTS

A total of 90 patients were found from April 2015 to October 2016, 83% (N=75) were female and 27% (N=15) were male, with mean age of 84 ± 7,73. Of these 20 patients had osteoarthritis of the knee; 29 patients had osteoarthritis of the hip, and 41 patients were admitted with a hip fracture.

Table I shows the mean serum levels of Vit D recorded in patients undergoing knee arthroplasty (N=20). Only female patients underwent knee arthroplasty, and showed low Vit D levels (mean value 16.8 ± 12.6 ng/ml in the preo-perative and mean value 18.2 ± 15.1 ng/ml in the post-operative period).

In Table II are recorded the mean serum level of Vit D in patients undergoing hip arthroplasty (N=29). Male subgroup (7% of this patients group, N=2) showed severe hypovitaminosis D (mean value <4.20 ng/ml). Female subgroup (93% of this patients group, N=27) exhibited mild hypovitaminosis with a slight decrease between pre and post-operative (mean value 25.2 ± 9.6 ng/ml and mean value 22.8 ± 9.7 ng/ml, respectively).

In Table III are recorded the mean serum level of Vit D in patients with proximal femoral fracture (N=41). Male subgroup (31.7% of this patients group, N=13) exhibited severe deficiency of Vit D (mean value 6.38 ± 5.68 ng/ml), with a evident increase in the post operative period (mean value 18.44 ± 4.63 ng/ml). Female subgroup (69.3% of this patients group, N= 28) showed a slight decrease of mean value of serum level of Vit D from a mean value of 12.24 ± 6.3 ng/ml pre-operatively to a mean value of 17.91 ± 19.3 ng/ml post-operatively.

## IV. DISCUSSION

This pilot study shows a high prevalence of Vit D deficiency in trauma and elective orthopaedics patients from Southern Italy. In patients with knee arthroplasty (Table I), all females, no Vit D deficiency was evident. In patients with hip osteoarthritis (Table II), the only two male patients had a moderately low mean Vit D serum level (<4.20 ng/ml). Female patients undergoing knee arthroplasty experienced an increase of almost 1.5 ng/ml in the 4 days following the index procedure, indicating a possible reduction of the inflammatory state, in addition to the resulting benefit from Vit D supplementation therapy. We would have expected the same raise in mean values even in patients undergoing hip arthroplasty. Instead, these values dropped. In male patients, the values were below the detection level of the assay. Females experienced a reduction of about 3.5 ng/ml, indicating a possible resistance to Vit D supplementation or a persistent inflammatory state. Patients with a hip fracture (Table III) gradually return towards normal Vit D levels already in the early post-operative period. This is especially evident in males, who seem more responsive to supplementation.

Reid et al. [18] showed that, as a result of knee arthroplasty, the serum levels of Vit D decreased. They measured Vit D and C reactive protein levels. From the second post-operative day, patients experienced a 40% reduction in Vit D and an increase in C-reactive protein: the levels did not normalize until 5 days after surgery, and the values remained low three months after surgery. Vit D levels fall following an inflammatory insult. Despite such decline in Vit D, it is unclear which specific mediators are responsible for the fall. Henriksen et al. [19] suggest that pro-inflammatory cytokines mediate Vit D decline. In thesis setting, cytokine and Vit D evaluations were performed before and after arthroplasty procedures. Mild decreases (12%) were observed before surgery and two days later, and a marked decrease (74%) from 3 weeks to 8 weeks after surgery, in line with the simultaneous increase in pro-inflammatory cytokines (TNF-α, IFN-γ, IL-1β, GM-CSF and IL-6) in serum.

Vit D modulates the activity of metalloproteinases, enzymes which are involved in the homeostasis of articular cartilage [20]. Low levels of 25(OH)D3 increase the production of degradative enzymes [21]. Low levels of Vit D slow down the remodeling response of subarticular bone, resulting in thickening of the subchondral bone, osteophyte formation and resultant cartilage and bone damage [22].

There are limitations to this study. We are, for example, aware that this is a pilot study, and that it only takes a snapshot of the complex tissue of Vit D metabolism. Ideally, the study should have continued with evaluation of Vit D levels at 3 months from surgery, but in our setting many patients do not attend for follow up. Also, pre-admission Vit D intake and sunlight exposure were not documented.

Compared to the above-mentioned studies [18,19], we enrolled a relatively large number of elderly patients. However, no weight and height measurement were documented, and it was therefore not possible to calculate the patients’ body mass index. The levels of inflammatory cytokines were not measured, and the C Reactive Protein was not taken into consideration. No α- or γ-tocopherol [23] or metalloprotease levels were measured [22].

Nevertheless, given the data collected, it is evident that even in a sunny country these patients exhibit a worryingly high prevalence one of Vitamin D deficiency. Therefore, it could be argued at prophylactic pre-hospital Vit D supplementation could be implemented [5], together with Vitamin C and E (α- or γ-tocopherol alone or in combination). These are potent dietary antioxidants which decrease cytokine (such as IL-6 and TNF-α) levels [23] to improve musculoskeletal health. These treatments could facilitate surgery and, above all, facilitate the functional recovery of the patient in the post operative phases.

This work is a pilot study, and opens up a series of interesting questions. For example, the biological mechanism through which cytokines induce the fall in serum levels of Vit D could be investigated, and ascertain how this fall could be prevented or reversed. It is possible that genetic predisposition is linked to Vit D deficiency, in fact, Conti et al.[24] investigated the effect of two single polymorphisms of the vitamin D receptor (VDR) gene in modulating bone mineral density (BMD) and the response to treatment with bisphosphonates or strontium ranelate in postmenopausal osteoporosis. The therapy response was different for the two types of receptor and for this the treatment should be personalized.

A genetic polymorphism of the Vit D receptor gene are associated with other orthopedic disease, for example the Taq I vitamin D receptor polymorphisms may be associated with abnormal acetabular morphology with increased of the risk of developing dysplasia of the hip or primary protusion acetabuli [25]. Also, it is not clear whether Vit D supplementation started post-operatively really restores optimal levels.

## V. CONCLUSIONS

Patients who are candidates for hip and knee arthroplasty and hip fracture patients exhibit a high rate of Vit D deficiency. In patients at risk for these pathologies, pre-hospital supplementation should be considered.

## Figures and Tables

**Table 1:** Mean serum Vit. D concentrarion in patients with osteoarthritis of the knee.

	Entire population	Female population	Male population
Pre-surgery	16.8 ± 12.6 ng/mL (N=20)	16.8 ± 12.6 ng/mL (N=20)	-
Fourth Day post-surgery	18.23 ± 15.1 ng/mL (N=18)	18.23 ± 15.1 ng/mL (N=18)	-

**Table 2:** Mean serum vit. D concentration in patients with osteoarthritis of the hip

	Entire population	Female population	Male population
Pre-surgery	21.86 ±11.4 ng/mL (N=29)	25.2 ± 9.6 ng/mL (N=27)	< 4.2 ng/mL (N=2)
Fourth Day post-surgery	20.2 ± 11.32 ng/mL (N=7)	22.8 ± 9.7 ng/mL (N=5)	< 4.2 ng/mL (N=2)

**Table 3:** Mean serum vit. D concentration in patients with proximal femur fracture

	Entire population	Female population	Male population
Pre-surgery	9.92 ± 6.28 ng/mL (N=41)	12.24 ± 6.3 ng/mL (N=28)	6.38 ± 5.68 ng/mL (N=13)
Fourth Day post-surgery	17.96 ± 18.47 ng/mL (N=32)	17.9 ± 19.3 ng/mL (N=21)	18.44 ± 4.63 ng/mL (N=11)
